# 

**Published:** 1947

**Authors:** 


| | Page |
|---|---|
| Back to Work. By H. Chitty, M.S., F.R.C.S. .. .. .. | 1 |
| The Treatment of Subacute Bacterial Endocarditis with Penicillin. By C. Bruce Perry, M.D., Ch.B., F.R.C.P. .. .. .. | 6 |
| Resection of the Head of the Pancreas. By F. D. Murphy, O.B.E., F.R.C.S. .. .. .. .. .. .. .. .. .. | 8 |
| Upper Parietal Meningioma showing Foster Kennedy Syndrome. By Diana J. Kinloch Beck, M.B., B.S., F.R.C.S., F.R.C.S.E. | 11 |
| Reviews of Books .. .. .. .. .. .. .. .. | 13 |
| Editorial Note .. .. .. .. .. .. .. .. | 16 |
| Meetings of Societies .. .. .. .. .. .. .. | 17 |
| The Medical Library of the University of Bristol .. .. .. | 18 |
| Publications Received .. .. .. .. .. .. .. | 19 |
| Local Medical Notes .. .. .. .. .. .. .. | 20 |

